# Remote Monitoring of Cardiovascular Implantable Devices in the Pediatric Population Improves Detection of Adverse Events

**DOI:** 10.1007/s00246-013-0774-5

**Published:** 2013-08-15

**Authors:** Lindsey E. Malloy, Jean Gingerich, Mark D. Olson, Dianne L. Atkins

**Affiliations:** 1 University of Iowa Children’s Hospital, Iowa City, IA 52242 USA; 2 Department of Pediatrics, Carver College of Medicine, University of Iowa, Iowa City, IA 52242 USA; 3 Division of Pediatric Cardiology, University of Iowa Hospital and Clinics, 200 Hawkins Drive, 2801 JPP, Iowa City, IA 52242 USA

**Keywords:** Remote monitoring, Pediatrics, Implantable cardiac devices, Home monitoring, Telemedicine, Remote follow-up

## Abstract

With the exponential growth of cardiovascular implantable electronic devices (CIEDs) in pediatric patients, a new method of long-term surveillance, remote monitoring (RM), has become the standard of care. The purpose of this study was to determine the usefulness of RM as a monitoring tool in the pediatric population. A retrospective review was performed of 198 patients at the University of Iowa Children’s Hospital who had CIEDs. Data transmitted by RM were analyzed. The following data were examined: patient demographics; median interval between transmissions; detection of adverse events requiring corrective measures, including detection of lead failure; detection of arrhythmias and device malfunctions independent of symptoms; time gained in the detection of events using RM versus standard practice; the validity of RM; and the impact of RM on data management. Of 198 patients, 162 submitted 615 RM transmissions. The median time between remote transmissions was 91 days. Of 615 total transmissions, 16 % had true adverse events with 11 % prompting clinical intervention. Of those events requiring clinical response, 61 % of patients reported symptoms. The median interval between last follow-up and occurrence of events detected by RM was 46 days, representing a gain of 134 days for patients followed-up at 6-month intervals and 44 days for patients followed-up at 3 month-intervals. The sensitivity and specificity of RM were found to be 99 and 72 %, respectively. The positive and negative predictive values were found to be 41 and 99 %, respectively. RM allows for early identification of arrhythmias and device malfunctions, thus prompting earlier corrective measures and improving care and safety in pediatric patients.

## Introduction

The use of cardiovascular implantable electronic devices (CIEDs)—including pacemakers, cardioverter defibrillators (ICDs), implantable loop recorders, and cardiac resynchronization therapy (CRT-D and CRT-P)—in both adults and children has grown exponentially. To ensure proper use, these devices require regular follow-up. To address this need, a new method of long-term surveillance, remote monitoring (RM), has been developed. The advent of RM systems for CIEDs offers many options and at the same time raises many questions regarding its implementation, organization of the obtained wealth of data, safety, legal issues, and reimbursement [8].

Home monitoring was first introduced in 1971 with transtelephonic monitoring of pacemakers. Within the last decade, several device manufacturers have introduced RM technology to enhance monitoring capabilities. RM uses the Internet as a means to interrogate CIEDs and download the stored information, thus allowing physicians to troubleshoot problems when they arise or before symptoms. As a result, patients may avoid extra clinic or emergency room visits. The development and use of RM has changed the standard for the management of patients with implanted devices. A recent expert consensus statement of the Heart Rhythm Society (HRS) and the European Heart Rhythm Society (EHRA) affirmed the earliest possible identification of abnormal device behavior, as well as the prevention of device malfunction underreporting, as being the primary goal of remote CIED monitoring [9].

Recent studies have shown that RM using the Internet is considered a milestone in the management of adult patients with an implantable cardiac device [6, 9]. Although the effectiveness of RM has been evaluated in adults, its usefulness and accuracy in the pediatric population has not been evaluated. Children are more prone to lead malfunctions; systems may be more complicated because of congenital heart disease; and it is challenging for young children to describe symptoms. Accordingly, RM may improve our ability to manage and care for these patients.

The purpose of this study was to determine the usefulness of RM as a monitoring tool in the pediatric population with CIEDs by (1) evaluating the adherence of pediatric patients with CIEDs to RM procedures; (2) evaluating early detection of adverse events prompting corrective measures, including detection of lead failure; (3) determining the ability of RM to detect arrhythmias and device malfunctions independent of symptoms; and (4) evaluating the validity of RM.

## Methods

We performed a retrospective review of all patients at the University of Iowa Children’s Hospital who had a CIED with RM capability between March 31, 2006, and October 14, 2011. Data transmitted by an RM system [Medtronic CareLink (Minneapolis, MN) and Boston Scientific Latitude (Natick, MA)], and the patient’s medical record were analyzed. This project was approved by the University of Iowa Institutional Review Board.

The following were examined: Patient demographics, median interval between remote transmissions; detection of adverse events prompting corrective measures; time gained in the detection of events using RM versus standard practice; sensitivity and specificity of RM; positive and negative predictive valve of RM; and the impact of RM on data management.

The types of events detected and transmitted during the study period were classified into groups with concerns related to disease/rhythm (sensed arrhythmias requiring therapy), device function (elective replacement indicator/end of life), and lead function (fracture, impedance changes, failure). Adverse events that prompted clinical response as defined by Chen et al. [1] are listed in Table 1. Clinical response was defined as an event requiring medication adjustment, pace termination, shock, cardioversion, generator replacement, or lead replacement.

**Table 1:** Clinically actionable events

Disease/rhythm-related	Detected atrial tachycardia/fibrillation in patients requiring pace termination, shock, cardioversion, or medication adjustment
	Detected sustained ventricular tachycardia ≥5 beats requiring pace termination, shock, cardioversion, or medication adjustment
	Asystole >3-s pause
Device function	Elective replacement indicator or end of life present
Lead function	Significant changes in atrial or ventricular lead impedance defined as impedance <200 or >2000 Ω, unstable lead impedance deemed to be clinically actionable, ≥50 % change in lead impedance since last interrogation
	Increase in pacing voltage threshold ≥1 V compared with the previous interrogation

The median interval between remote transmissions was calculated and compared with the recommended follow-up guidelines. Only one remote transmission per day was included in the median interval calculation. The median time interval between first report of an event and last device interrogation in the clinic was calculated. Assuming the typical protocol at our institution of twice-yearly in-person follow-up schedule for pacemaker, ICD, and CRT system surveillance and quarterly follow-up for loop recorders, the time gained in the detection of events using RM versus standard practice was calculated as 180 − X days and 90 − X days, respectively. Sensitivity was calculated as follows: number of true-positive events/(number of true-positive + false-negative events). Specificity was calculated as follows: number of true-negative events/(number of true-negative + false-positive events). Positive predictive value was calculated as follows: number of true-positive events/(number of true-positive + false-positive events). Negative predictive value was calculated as follows: number of true-negative events/(number of true-negative + false-negative events). True-positive events were defined as patients with an event who were correctly diagnosed as having an event. False-positive events were defined as patients without an event who were incorrectly identified as having an event. True-negative events were defined as patients without an event who were correctly identified as not having an event. False-negative events were defined as patients with an event who were incorrectly identified as not having an event. The impact of RM on data management was examined by evaluating the number of remote transmissions from 2006 to 2011 and by calculating the average number of reports received per week and per month.

## Results

Between March 31, 2006, and October 14, 2011, 198 patients had CIEDs with RM capability, including the following: pacemakers (*n* = 105), cardioverter defibrillators (*n* = 61), implantable loop recorders (*n* = 27), combined cardiac resynchronization therapy pacemaker systems (*n* = 2), and combined cardiac resynchronization therapy ICD systems (*n* = 3). Primary diagnoses included the following: complete heart block 38 % (*n* = 76), sinus node dysfunction 16 % (*n* = 32), ventricular arrhythmias 14 % (*n* = 27), syncope 8 % (*n* = 16), long QT syndrome 7 % (*n* = 13), atrial arrhythmias 6 % (*n* = 11), atrial and ventricular arrhythmias 3 % (*n* = 6), asystole 2 % (*n* = 5), and other 6 % (*n* = 12). Forty-nine percent (*n* = 97) of patients included in the study had congenital heart disease. The average age of patients was 21 ± 12 years (SD), and the median age was 18 years (range 1–63). There were 100 male and 98 female patients.

**Fig. 1:**
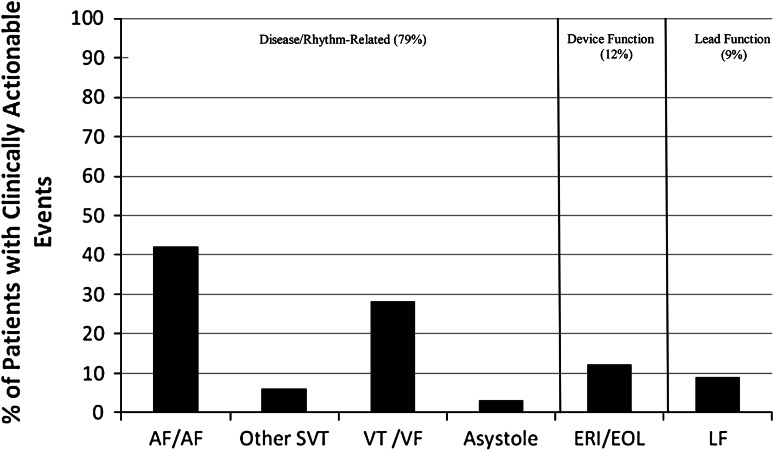
Percentage of patients with clinically actionable events transmitted by RM services according to clinical concerns. *AF*/*F* atrial fibrillation/flutter, *SVT* supraventricular tachycardia, *VT*/*VF* ventricular tachycardia/ventricular fibrillation, *ERI*/*EOL* elective replacement indicator/end of life, *LF* lead failure

**Fig. 2:**
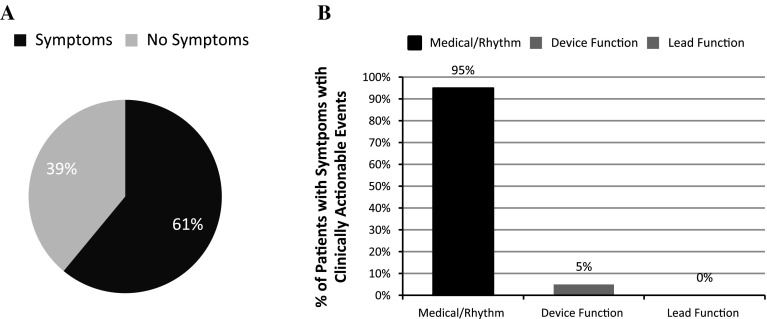
**a** Percentage of patients with symptoms versus no symptoms associated with a clinically actionable remote transmission. **b** Percentage of patients with symptoms grouped by concerns related to disease/rhythm, device function, and lead function

Of the 198 total patients with CIEDs during the study period, 18 % (*n* = 36) did not have a remote transmission recorded. The median time between remote transmissions was 91 days (range 1–842). Of the 615 transmissions, 461 were remote transmissions for routine follow-up. One hundred fifty-four transmissions were for a specific indication (patient symptoms, device alarm). Thirteen percent (60 of 461) of remote transmissions for routine follow-up were found to be true events, whereas 27 % (42 of 154) of remote transmissions for a specific indication were found to be true events. Sixteen percent of total transmissions (*n* = 615) detected true adverse events (*n* = 101), and 11 % (*n* = 65) of these prompted clinical intervention. Of the transmissions with true adverse events, 64 % (*n* = 65) prompted clinical intervention. Of the patients requiring clinical intervention, 79 % (*n* = 51) had disease/rhythm-related concerns, 12 % (*n* = 8) had general device status concerns, and 9 % (*n* = 6) had lead failure concerns (Fig. 1). Of those with events detected by RM, 61 % of patients had symptoms (*n* = 40), and 39 % (*n* = 25) had no symptoms associated with the event (Fig. 2a). Of the patients with symptoms, 95 % (*n* = 38) were associated with concerns related to disease/rhythm (sensed arrhythmias requiring therapy); 5 % (*n* = 2) were associated with device function (elective replacement indicator/end of life); and none were associated with lead function (fracture, impedance changes, failure) (Fig. 2b). For patients receiving a shock, 13 patients sent a specific transmission for symptoms. One patient was noted to have a shock on routine RM who was unaware of the shock.

Of the patients (*n* = 13) who were included in the Sprint Fidelis lead (Medtronic Inc., Minneapolis, MN) recall [2], 9 had RM capability. Two patients were found to have lead failure on RM before an inappropriate shock was delivered. One patient had a remote transmission sent ~3 months previously, whereas the other patient had a clinic appointment ~2 months before lead failure both of these showed normal functioning leads. One patient received an inappropriate shock related to lead failure and did not had RM capability. No patients received an inappropriate shock related to lead failure that had RM capability.

The median interval between last follow-up and occurrence of events detected by RM was 46 days (range 1–467), representing a temporal gain of 134 days for patients followed-up at 6-month intervals and 44 days for patients followed-up at 3-month intervals. One patient complained of palpitations, later found to be atrial flutter, and the device did not detect an arrhythmia because of far-field oversensing (<0.2 %). One transmission showed an inappropriate shock when no arrhythmia was present (<0.2 %). The sensitivity of detecting arrhythmias or device problems was found to be 99 % (101 of 102), whereas the specificity was found to be 72 % (369 of 513). The positive predictive value of RM was found to be 41 % (101 of 245), whereas the negative predictive value was 99 % (369 of 370). There were 144 false-positive remote tracings identified, mostly secondary to sinus tachycardia being erroneously identified as a tachyarrhythmia. Five transmissions showed that the device was not successful at terminating the event, and further medical management was needed (0.8 %). To further distinguish between remote transmissions for routine follow-up compared with those for specific indications, the sensitivity of routine RM was found to be 100 % (60 of 60), whereas the specificity was found to be 77 % (309 of 401). The sensitivity of RM for a specific indication was found to be 98 % (42 of 43), whereas the specificity was found to be 54 % (60 of 111). The positive predictive value of routine RM was found to be 39 % (60 of 152), whereas the negative predictive value was 100 % (309 of 309). The positive predictive value of RM for specific indications was found to be 45 % (42 of 93), whereas the negative predictive value was 98 % (60 of 61).

Of the 36 patients (18 %) who did not submit remote transmissions, 16 (44 %) were noncompliant; 5 (14 %) did not receive insurance reimbursement; 4 (11 %) did not have access to a land-line telephone; 1 (3 %) died; and 10 (28 %) for because of unknown causes. Of the patients who did not submit because of unknown causes, 5 (50 %) had a loop-recorder device.

**Fig. 3:**
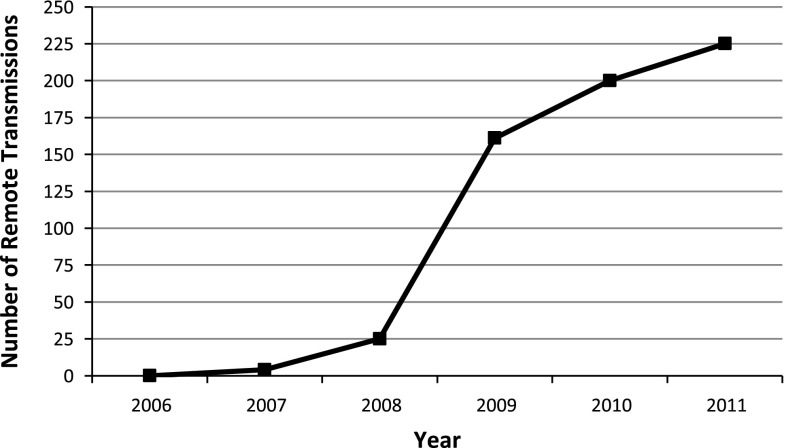
Number of remote transmissions from 2006 to 2011

In 2007, there were 4 transmissions; in 2008 there were 25 transmissions; in 2009 there were 161 transmissions; in 2010 there were 200 transmissions; and in 2011 there were 225 transmissions (Fig. 3). In 2011, there was a mean of 0.6 remote transmissions/day, equating to ~4 transmissions/week. Time spent by the pacemaker nurse practitioner with analyzing and documenting transmissions averages 30 min for a noncomplicated pacemaker, ICD, and loop-recorder interrogation. For more complicated interrogations, the time spent varies and can take ≤1 h.

## Discussion

RM is designed for early identification of arrhythmias and device malfunctions, which can ultimately prompt earlier corrective measures and improve care and safety in pediatric patients. Our study shows that both goals are achieved in the pediatric population. Of 615 total transmissions, 16 % had true adverse events, 11 % of which prompted clinical intervention. Of those events requiring clinical response, 39 % of patients did not have clinical symptoms. The median interval between last follow-up and occurrence of events detected by RM was 46 days, representing a gain of 134 days for patients followed-up at 6-month intervals and 44 days for patients followed-up at 3-month intervals. Two patients were found to have lead failure on RM before an inappropriate shock was delivered. No patients received an inappropriate shock related to lead failure that had RM capability.

RM allows for storage of large amounts of data regarding device function, diagnostics, delivered therapy, and intracardiac hemodynamics [3]. Obtaining and evaluating these data in a timely manner requires the cooperation of both the provider and the patient. Patient compliance with sending remote transmissions compared with standard guidelines was evaluated. Our data show that the median time between transmissions for all CIEDs was approximately 3 months. The standard guidelines published by the HRS/EHRA consensus paper recommend that a minimum interval of CIED RM should be 3–12 months for pacemakers and CRT-P, 3–6 months for ICD/CRT-D, and 1–6 months for implantable loop recorders [9]. Our study shows that pediatric patients with CIEDs appropriately adhere to RM procedures. We found that only 18 % of patients never submitted a remote transmission. Although most of this was accounted for by patient noncompliance, some was due to the refusal of the insurance company to reimburse for this expense as well as the unavailability of a land-line telephone. Technological improvements of RM, especially as use of land-line telephones decreases may improve compliance. Those that do not comply have a much greater chance of later detection of arrhythmias and device malfunctions that may lead to increased morbidity and mortality.

We found that using RM affords a temporal gain of 134 days from last follow-up to occurrence of an event for patients followed-up at 6-month intervals. These data are similar to the AWARE data, which found a decrease of 154 days, and the COMPAS data, which found a decrease of 144 days compared with conventional 6-month follow-up in the adult population [4, 5]. More importantly, earlier detection of device and/or lead malfunction may improve patient safety because these were found to be associated with patient symptoms only 5 % of the time and none of the time, respectively. This is especially important in the pediatric population where children’s ability to describe symptoms and interpretation of their descriptions may be challenging. For example, our study found that two patients had lead failure on RM included in the lead recall before an inappropriate shock was delivered. In addition, RM detected a shock delivered for ventricular fibrillation during the night in a patient who was not aware of receiving any therapy. RM was able to prevent an inappropriate shock and to detect an appropriate shock in an asymptomatic patient, thus improving the care and safety of pediatric patients.

In addition, our data suggest that RM can serve as a reliable monitoring tool in providing pediatric patients, their families, and physicians with a comfort level regarding evaluation of device or patient issues when concerns do arise. We found that 99 % of the time, if there is no alert present on RM, the patient is not having a concerning problem or device issue. Therefore, with RM in place, patients and their families can feel confident that if no alert is present, neither the device nor the patient is currently experiencing problems. In cases where the alert indicates a concerning device or patient problem, our data show that only 41 % of the time this is a true concerning device or patient problem. Consequently, RM can serve as a monitoring tool to help rule out concerning events; however, this must be analyzed closely to rule in concerning events. When comparing routine RM versus RM for specific indications, no difference was seen in sensitivity, positive or negative predictive value. There was a decrease in the specificity of RM for a specific indication probably secondary to an increase in false-positive transmissions.

RM has the potential to allow for fewer clinic visits of the rapidly growing and more mobile patient population [7]. The HRS/EHRA consensus paper on CIEDs suggests that it is safe to decrease the number of clinic visits for ICD and CRT-devices to annually provided that a transmission is submitted every 3–6 months [9]. Decreased number of clinic visits is certainly desirable for patient and physician time management and may prove to be cost-effective. Our study shows that patients are appropriately compliant with routine transmissions, thus providing physicians with reassurance that patients will not likely go an entire year without follow-up. Only 14 % of patients (*n* = 23) went >1 year without sending a remote transmission. Our study also showed that RM is a reliable monitoring tool for evaluating patient and/or device concerns and also showed that problems were detected weeks to months before face-to-face encounters would have detected a problem. This suggests that it could be safe to decrease the number clinic visits for a subset of patients with CIEDS from every 6 months to annually provided they are compliant with RM.

The now “virtual patient” created by RM creates a paradigm shift. Physician practices have the responsibility of responding to these new sources of patient data, creating appropriate documentation for reimbursement and scheduling future device downloads [6]. With the increased use of RM, methods are needed to manage the flow of information. It remains to be well-documented how much time is needed to deal with these alerts. With the increase in CIED implantations over the recent years, our study showed that there has been an exponential increase in the number of remote transmissions. Since 2009, the number of remote transmissions has increased by an average of 32 transmissions/year, representing a 14 % increase in 2011. There are currently no practice guidelines on the role of remote follow-up addressing the previously mentioned concerns. However, there is a need for an infrastructure and a protocol to address the issue of these notifications as the volume of remote data continues to increase. Manpower, operational and organization flow is an area that requires further exploration and analysis. Reimbursement and cost-effectiveness have not been thoroughly evaluated, and this is beyond the scope of this article. Future studies are certainly needed to address these important issues.

Some study limitations that should be noted. This study addressed only those patients with CIEDs who sent a remote transmission. Documentation of why patients did not send remote transmissions could not always be obtained. Patient compliance with RM may limit the number of transmissions and accuracy of the data. Patient recall and reporting of symptoms may not be accurate and lead to recall bias.

## Conclusion

RM allows for early identification of arrhythmias and device malfunctions, thus prompting earlier corrective measures in pediatric patients. Families are compliant with the use of the technology. RM can improve the care and safety of pediatric patients.
